# Case report: Deep molecular remissions post two separate CD19-targeted chimeric antigen receptor T-cell therapies do not prevent disease from relapsing in Philadelphia chromosome-positive acute lymphoblastic leukemia

**DOI:** 10.3389/fonc.2023.1251738

**Published:** 2023-11-03

**Authors:** Yu Tang, Xiaoming Fei, Xianqiu Yu, Jiang Cao, Lixia Wang, Fang Lei

**Affiliations:** ^1^ Department of Rheumatology and Immunology, Affiliated Hospital of Jiangsu University, Zhengjiang, Jiangsu, China; ^2^ Department of Hematology, Affiliated Hospital of Jiangsu University, Zhengjiang, Jiangsu, China; ^3^ Department of Hematology, Affiliated Hospital of Xuzhou Medical University, Xuzhou, Jiangsu, China

**Keywords:** acute lymphoblastic leukemia, Philadelphia chromosome–positive, chimeric antigen receptor T-cell, allogeneic, stem cell transplantation

## Abstract

Philadelphia chromosome–positive acute lymphoblastic leukemia (Ph+ ALL) is an aggressive B-cell malignancy. The management of a relapsed Ph+ ALL patient is challenging. Currently, either allogeneic stem cell transplant (allo-SCT) or CD19-targeted chimeric antigen receptor T-cell (CAR T-cell) are usually employed as salvage modalities for a relapsed patient. However, there are few reports concerning cases that had both allo-SCT and multiple CAR T-cell therapies, and the optimal management of such patients is unclear. Here, we report a relapsed Ph+ ALL male who was first salvaged with autologous CAR T-cell therapy, followed by allo-SCT. Unfortunately, he had a second relapse even with complete molecular remission (CMR) response after the first CAR T and allo-SCT. This patient was then successfully salvaged by a second CAR T-cell product that is donor-derived. However, even with a CMR response once again following the second CAR T-cell therapy and prophylactic donor lymphocyte infusion, he experienced a molecular relapse; ponatinib was employed as the subsequent salvage treatment. He achieved a CMR response following ponatinib and was still in remission at the last follow-up. No ABL kinase mutation was detected during the whole course of the disease. This case indicated that a repeated CD19-targeted CAR T-cell treatment is feasible and may be effective in a relapsed Ph+ ALL patient that had previous CAR T-cell and allo-SCT, even though both CAR T-cell have the same construction. However, even with a deep response after each CAR T-cell therapy and allo-SCT, there is still a very small amount of undetectable leukemic cells. The optimal management of Ph+ ALL patients who have a deep response after a second CAR T-cell therapy deserves further exploration.

## Introduction

Philadelphia chromosome–positive acute lymphoblastic leukemia (Ph+ ALL) accounts for approximately 25% of adult ALL cases and close to 50% of cases in older adults (1). Patients with Ph+ ALL have a dismal outcome when treated with chemotherapy alone. The 5-year overall survival (OS) rate is poor, usually less than 10% (2). Even with an intensive therapeutic regiment such as hyper-CVAD, the 5-year OS is just 12% (3). The addition of tyrosine kinase inhibitors (TKIs) to chemotherapy significantly improves OS. Jabbour et al. reported that the combination of ponatimib with hyper-CVAD resulted in a 3-year event free survival of 70% (95% CI 56–80) (4). For relapsed Ph+ ALL cases who had been previously treated with TKIs plus chemo, chimeric antigen receptor T-cell (CAR T-cell) therapy and allogeneic stem cell transplant (allo-SCT) are among the preferred salvage options (5, 6). However, how to manage a Ph+ ALL patient that already had chemo plus TKIs, allo-SCT, and CAR T-cell therapy is still unclear. Here, we present a relapsed Ph+ ALL patient who underwent allo-SCT and two separate CD19-targeted CAR T-cell therapies pre- and post-transplant, and this patient experienced multiple relapses in spite of a deep response to all the salvage therapies.

## Case report

**Figure 1:**
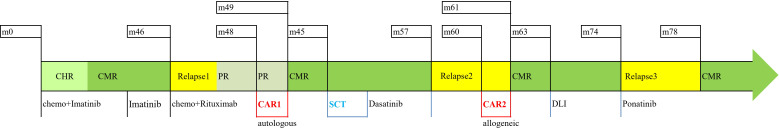
Summary of the treatment. Chemo, chemotherapy; CAR1, first CAR T-cell therapy; SCT, stem cell transplantation; CAR2, second CAR T-cell therapy; DLI, donor lymphocyte infusion; CHR, complete hematological remission; CMR, complete molecular remission; PR, partial remission.

A 45-year-old male initially presented with fatigue and fever in October 2015 and he was found to have a high WBC count together with anemia and thrombocytopenia. A bone marrow (BM) exam was consistent with the diagnosis of precursor B-cell ALL: 95% blast with an immunophenotype of CD19+, CD20+, CD10+, CD22+, CD34+, and TdT+. A cytogenetic test found t (9; 22)(q34; q11) and further molecular testing confirmed the presence of BCR/ABL1 (coding for a 190-kDa protein). He initially received an induction of imatinib plus VDCP (vincristine, daunorubicin, cyclophosphamide, and predonisone), and achieved complete hematological remission at the end of induction. Subsequently, he received multiple cycles of imatinib plus chemotherapy for consolidation. Complete molecular remission (CMR) at the level of 10^−6^ was achieved after two courses of chemotherapy. He had imatinib maintenance after the completion of consolidation and maintained CMR until the first hematological relapse was documented in July 2019. At the time of first relapse, a BM exam showed 85% blast with a similar immunophenotype to that at diagnosis. An ABL kinase domain mutation test was negative. The patient received two courses of rituximab+ hyper-CVAD salvage and achieved partial remission (PR). We enrolled him into a CAR T-cell therapy clinical trial (ClinicalTrials.gov # NCT0278235), and an autologous humanized CD19-target CAR T-cell product was manufactured as previously described (7, 8). He received FC (fludarabine plus cyclophosphomide) lymphodepletion prior to the infusion of 1.8 × 10^6^ CAR T-cells per kilogram of body weight on 30 September 2019 and developed grade I cytokine release syndrome (CRS) after CAR T-cell infusion. A CMR response was confirmed 1 month after the first CAR T-cell infusion. He received no additional treatment thereafter and remained CMR. In March 2020, he underwent related haploindentical allo-SCT, employing a modified T-cell-replete GIAC protocol (9) plus low-dose post-transplant cyclophosphamide (PTCY) for graft versus host disease (GVHD) prophylaxis (10). Neutrophil and platelet engraftment was achieved at +16 and +19 days, respectively. Chimerism testing showed ≥ 99% of donor origin at +1, +2, and +3 months. The patient developed grade I acute GVHD and grade I chronic GVHD after allo-SCT. He then received dasatinib maintenance post-SCT and multiple MRD tests were all negative until April 2021. A low level of BCR-ABL1 transcript (at the level of 10^−3^) was detected, which indicated a molecular relapse of the disease. Donor-derived lymphocyte was harvested from the patient himself by leukapheresis, and a CD19-target CAR T-cell product with the same construct as the previous one was manufactured. In May 2021, the second CAR T-cell product was infused at a dose of 1.0 × 10^6^ CAR T-cells per kilogram of body weight. Immediately before the second CAR T-cell infusion, a BM exam showed 21% of blasts with a CD19+ immunophenotype. The patient received no GVHD prophylaxis and developed grade I CRS following the second CAR T-cell infusion. MRD-negative remission was documented at +1 month post-CAR T-cell infusion. To further minimize the rate of relapse, a prophylactic escalating dose regimen of donor lymphocytes infusion was performed from August 2021 to May 2022. However, a low level of BCR/ABL1 transcript (8×10^−4^) was once again detected in June 2022; therefore, ponatinib was administrated as the treatment for molecular reemergence of residue leukemia. The BCR/ABL1 transcript began to drop on ponatinib and was below the detection limit (10^−6^) in October 2022. This patient is still on ponatinib treatment now and remained MRD negative at the last follow-up evaluation in January 2023. A brief summary of the treatment for this patient is illustrated in **Figure 1**.

## Discussion

Since the incorporation of TKIs into upfront Ph+ ALL management, the survival outcomes have substantially improved (2, 4, 5). Although some patients treated with the first and second generation may relapse with the emergence of a T315I clone (11), the introduction of ponatinib can effectively overcome this mutation (4). For this patient, who experienced multiple relapses while on TKI, since no ABL1 kinase mutations could be detected, it is difficult to predict if an in-class switch of TKI may have any effects. Based on previous reports, CD19-target CAR T-cell salvage was shown to have very high response rates in the relapsed/refractory B-ALL setting (6, 7); thus, CAR T-cell salvage should be a better option than in-class switch of TKI. For this patient, MRD-negative status was achieved after the first post-CAR T-cell therapy. MRD status at transplant was reported to be an independent factor associated with transplant outcome if a CAR T-cell salvaged patient subsequently underwent allo-SCT (12). Zhao et al. also reported that, for CAR T-cell salvaged relapsed/refractory ALL patients, those who subsequently received a transplant had a better 2-year OS and LFS than those who were not transplanted (12). To further minimize the rate of relapse, this patient undertook allo-SCT in spite of MRD negativity. However, it is impossible to verify if the SCT procedure further decreased the residue leukemic pool in this patient because all MRD tests showed negative results both post-CAR T-cell and post-SCT. Unfortunately, undetectable residue leukemic cells did result in a second relapse even with CAR T-cell therapy, allo-SCT, and dasatinib maintenance in this patient. Management of this type of relapsed Ph+ ALL patient is very challenging. Although some B-ALL patients who were pre-treated with a murine-derived product might have responded to a humanized CAR T-cell construct (13, 14), attempts to retreat B-ALL patients who had relapsed or had non-responded disease after the first CAR T-cell with the same CAR T construct were largely unsuccessful, with a CR rate of only 21% and a median duration of response of just 4 months (15). In another report on three allo-SCT recipients who received autologous CAR T-cell salvage prior to transplant, all three cases had post-transplant relapsed B-ALL and were treated with a second donor-derived CAR T-cell; only one patient achieved CR after CAR T2 (16). Fortunately, this patient had a complete response to the second CAR T product with an identical construct to the first CAR T.

To prepare CAR T-cell products for B-ALL patients who have a post-SCT relapse, allogeneic lymphocytes are usually collected from the donor (12, 17). Compared with the dose of T-lymphocyte infusion for DLI, the dose of T-cell infusion in CAR T-cell therapy is generally low (12, 15–17). However, GVHD sometimes did occur after donor-derived CAR T-cell infusion, although the incidence of GVHD is generally lower than that in the DLI setting (< 10% of all cases) (16, 17). In terms of the source of T cells in this patient, we highlight a unique point in that the second CAR T product was collected from the recipient himself; its efficacy is similar to the first CAR T-cell product prepared from autologous T cells. In addition to its efficacy, no GVHD occurred after allogeneic CAR T-cell infusion. Whether a donor-derived or recipient-derived CAR T-cell product is better is still an open question, although there is a report indicating that a better outcome may be associated with recipient-derived CAR T-cells (18).

Another unique point in this relapsed Ph+ ALL patient is that there is still a small amount of undetectable leukemic cells remaining after two separate novel CAR T-cell therapies, TKIs, and allo-SCT. This patient once again experienced molecular relapse even with a second CAR T-cell and DLI; however, MRD was negative after the initiation of ponatinib. As for the reason of MRD conversion, it is difficult to attribute it solely to ponatinib or DLI, although the MRD level began to decrease after ponatinib initiation. Recently, other agents, such as blinatumomab and inotuzumab ozogamicin, have been shown to be effective in the treatment of MRD-positive B-ALL. Although a successful conversion of MRD negativity was observed with the addition of ponatinib, to the best of our knowledge, there is no study to guide us on the choice of modalities to manage a heavily treated Ph+ ALL patient like the one in our study. It is now an unmet clinical need as an increasing number of Ph+ ALL patients are successfully salvaged by novel CAR T-cell therapies with different targets and constructs. A well-designed clinical trial concerning preventative strategies of relapse may solve this question in Ph+ ALL patients who have a CMR response after CAR-T cell salvage.

In conclusion, either an autologous or recipient-derived allogeneic CAR T-cell product are equally effective in the treatment of relapsed Ph+ ALL. Moreover, an allogeneic CAR T-cell product even works in a relapsed Ph+ ALL patient that had previous CAR T-cell therapy with an identical construct. However, MRD-negativity after each separate CAR T-cell therapy does not mean an elimination of leukemic cells. Therefore, in a patient like this one, a prophylactic regimen should be implemented immediately after the second CAR T-cell therapy, even with MRD negativity. Optimal prophylactic measures remain to be explored since there are many effective modalities such as bi-specific antibodies, third generation TKIs, antibody-drug conjugates, DLI, and different combinations and possible sequences.

## Data availability statement

The original contributions presented in the study are included in the article/supplementary material. Further inquiries can be directed to the corresponding author.

## Ethics statement

The studies involving humans were approved by Ethics Board of Affiliated Hospital of Jiangsu University. The studies were conducted in accordance with the local legislation and institutional requirements. The participants provided their written informed consent to participate in this study. Written informed consent was obtained from the individual(s) for the publication of any potentially identifiable images or data included in this article.

## Author contributions

Treatment decision-making and discussions: YT and XF. Data collection and analysis: XY, LW, and FL. CAR T-cell manufacture: JC. Manuscript writing: YT. Final approval of manuscript: XF. All authors contributed to the article and approved the submitted version.
